# Integrative In Silico and In Vivo Analysis of Banhasasim-Tang for Irritable Bowel Syndrome: Mechanistic Insights into Inflammation-Related Pathways

**DOI:** 10.3390/ph18081123

**Published:** 2025-07-27

**Authors:** Woo-Gyun Choi, Seok-Jae Ko, Jung-Ha Shim, Chang-Hwan Bae, Seungtae Kim, Jae-Woo Park, Byung-Joo Kim

**Affiliations:** 1 Department of Longevity and Biofunctional Medicine, School of Korean Medicine, Pusan National University, Yangsan 50612, Republic of Korea; ak0510@hanmail.net (W.-G.C.); luckydunk@naver.com (J.-H.S.); 2 Department of Clinical Korean Medicine, Graduate School, Kyung Hee University, Seoul 02447, Republic of Korea; kokokoko119@daum.net; 3 Department of Gastroenterology, College of Korean Medicine, Kyung Hee University, Seoul 02447, Republic of Korea; 4 Department of Korean Medical Science, School of Korean Medicine, Pusan National University, Yangsan 50612, Republic of Koreakimst@pusan.ac.kr (S.K.)

**Keywords:** Banhasasim-tang, irritable bowel syndrome, network pharmacology, molecular docking, molecular dynamics

## Abstract

**Background/Objectives:** Banhasasim-tang (BHSST) is a traditional herbal formula commonly used to treat gastrointestinal (GI) disorders and has been considered a potential therapeutic option for irritable bowel syndrome (IBS). This study aimed to explore the molecular targets and underlying mechanisms of BHSST in IBS using a combination of network pharmacology, molecular docking, molecular dynamics simulations, and in vivo validation. **Methods:** Active compounds in BHSST were screened based on drug-likeness and oral bioavailability. Potential targets were predicted using ChEMBL, and IBS-related targets were obtained from GeneCards and DisGeNET. A compound–target–disease network was constructed and analyzed via Gene Ontology and KEGG pathway enrichment. Compound–target interactions were further assessed using molecular docking and molecular dynamics simulations. The in vivo effects of eudesm-4(14)-en-11-ol, elemol, and BHSST were evaluated in a zymosan-induced IBS mouse model. **Results:** Twelve BHSST-related targets were associated with IBS, with enrichment analysis identifying TNF signaling and apoptosis as key pathways. In silico simulations suggested stable binding of eudesm-4(14)-en-11-ol to TNF-α and kanzonol T to PIK3CD, whereas elemol showed weak interaction with PRKCD. In vivo, eudesm-4(14)-en-11-ol improved colon length, weight, stool consistency, TNF-α levels, and pain-related behaviors—effects comparable to those of BHSST. Elemol, however, showed no therapeutic benefit. **Conclusions:** These findings provide preliminary mechanistic insight into the anti-inflammatory potential of BHSST in IBS. The integrated in silico and in vivo approaches support the contribution of specific components, such as eudesm-4(14)-en-11-ol, to its observed effects, warranting further investigation.

## 1. Introduction

Irritable bowel syndrome (IBS) is a common functional gastrointestinal (GI) disorder characterized by recurrent abdominal pain and altered bowel habits. It affects approximately 11% of the global population [1] and significantly impairs patients’ quality of life [2]. The pathogenesis of IBS is multifactorial, involving psychological stress, gut microbiota imbalance, intestinal dysmotility, visceral hypersensitivity, and prior infections [3]. Although IBS does not cause structural damage to the GI tract, it leads to persistent discomfort, reduced quality of life, and a substantial healthcare burden [4,5,6].

Since its emergence in 2007, network pharmacology has become a valuable approach in drug discovery, offering insights into polypharmacology and the complexity of biological systems [7]. By integrating computational tools, network pharmacology enables the analysis of protein–protein interactions, compound–target–disease (CTD) networks, and the identification of key therapeutic pathways, particularly in traditional medicine. Given that herbal formulas typically contain multiple bioactive compounds, this systems-level approach is especially useful for elucidating their mechanisms of action from a holistic perspective. For example, Zhou and Wu demonstrated that the mitogen-activated protein kinase (MAPK) pathway is a potential target of *Eucommia ulmoides* cortex in the treatment of osteoporosis [8]. Similarly, Dou et al. reported that tanshinone II, a compound from *Salvia miltiorrhiza* Bunge, exerted protective effects in an acute kidney injury model by activating the pregnane X receptor [9].

Banhasasim-Tang (BHSST), known as Hange-shashin-to in Japan, and an-xia-xiexin-tang in China, is a traditional herbal medicine formula composed of Coptidis Rhizoma (CR), Ginseng Radix (GR), Glycyrrhizae Radix et Rhizoma (GRR), Pinelliae Tuber (PT), Scutellariae Radix (SR), Zizyphi Fructus (ZF), Zingiberis Rhizoma (ZR), and Zingiberis Rhizoma Recens (ZRR). BHSST is primarily used for treating GI disorders such as dyspepsia, gastric ulcers, pharyngitis, colitis, diarrhea, gastroesophageal reflux disease, and esophageal ulcers caused by chronic reflux [10,11,12,13,14,15,16,17]. Additionally, BHSST is an effective mouthwash for treating oral mucosal inflammation and reducing PGE2 concentration in oral keratinocytes [18,19]. Moreover, it functions as an anti-inflammatory, antioxidant, and antimicrobial agent by inhibiting the COX-2 activity related to inflammation [19,20,21,22,23].

Although IBS has traditionally been considered a functional disorder, recent studies suggest that mucosal inflammation and immune activation may contribute to its pathogenesis [24]. Increased numbers of mast cells and other immune cells in the gut mucosa can release pro-inflammatory cytokines such as TNF-α and IL-6, leading to visceral hypersensitivity and altered motility [25]. Post-infectious IBS further supports the role of persistent inflammation in symptom development, possibly mediated through gut microbiota changes and gut–brain axis dysregulation [26].

Although traditional use and various studies—including our previous experimental research demonstrating the efficacy of BHSST in IBS models [27]—suggest a potential benefit of herbal medicines in IBS management [15,28,29], mechanistic evidence remains limited. Given the complexity of BHSST, which contains numerous phytochemicals, fully elucidating its mechanism of action is not feasible. Therefore, we adopted a representative compound-based approach to explore potential molecular interactions.

In this study, we employed network pharmacology and in silico simulations to predict interactions between selected active compounds in BHSST and IBS-related targets. Among them, eudesm-4(14)-en-11-ol and kanzonol T were identified as potential modulators of inflammatory pathways associated with IBS pathophysiology. To support these computational predictions, we conducted in vivo experiments using a zymosan-induced IBS mouse model. Eudesm-4(14)-en-11-ol significantly alleviated colon shortening, abnormal colon weight gain, altered stool consistency, elevated TNF-α levels, and pain-related behaviors. In contrast, elemol did not exhibit therapeutic efficacy.

While we acknowledge the limitations of extrapolating these findings to the full BHSST formulation or to human IBS, our results offer preliminary mechanistic insights into how individual components—particularly eudesm-4(14)-en-11-ol—may contribute to the observed effects of BHSST in IBS.

## 2. Results

### 2.1. Screening of Active Compounds in BHSST

**Figure 1:**
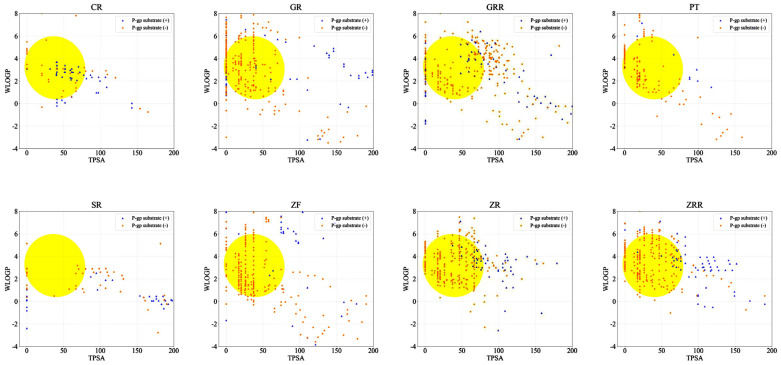
Predicted plots of the intestinal absorption and P-glycoprotein substrate potential of individual herbs in Banhasasim-tang. Compounds within the white ellipses indicate the human intestinal absorption potential. In particular, compounds within the yellow ellipse are predicted to cross the blood–brain barrier. Orange dots represent negative substrates for P-glycoprotein, whereas blue triangles denote positive substrates that are expected to be effluxed by P-glycoprotein. TPSA, topological polar surface area; CR, Coptidis Rhizoma; GR, Ginseng Radix; GRR, Glycyrrhizae Radix et Rhizoma; PT, Pinelliae Tuber; SR, Scutellariae Radix; ZF, Zizyphi Fructus; ZR, Zingiberis Rhizoma; ZRR, Zingiberis Rhizoma Recens.

**Figure 2:**
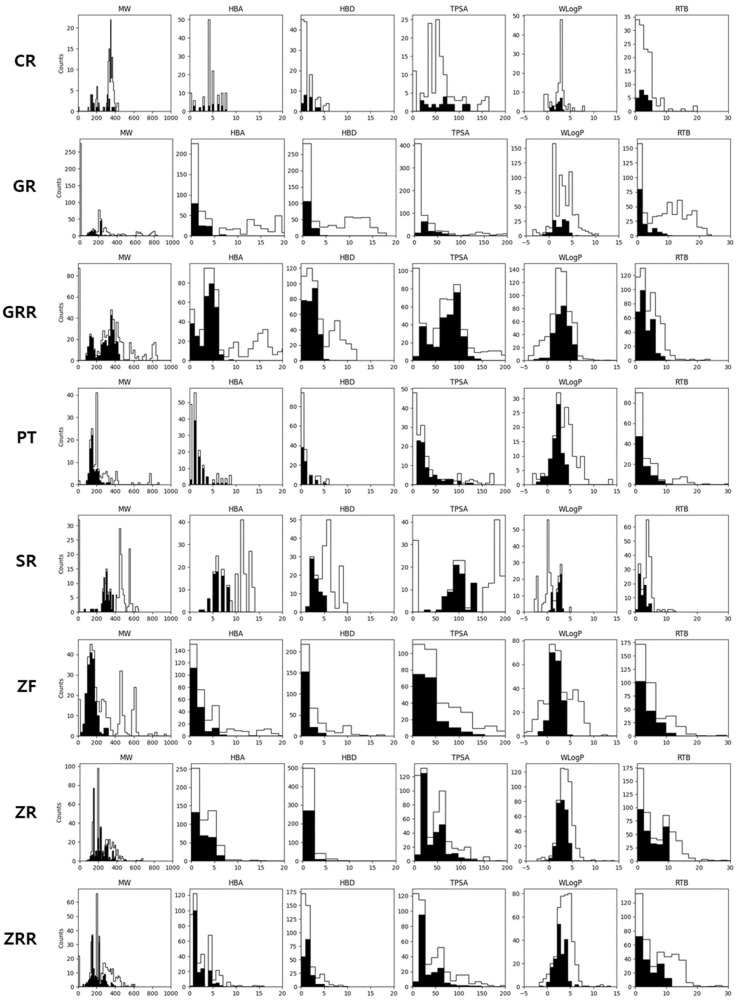
Histograms of the physicochemical properties of the active compounds in each BHSST formulation. The white histograms depict the distribution of all compounds within the formulation, whereas the black histograms illustrate the distribution of active compounds that meet the drug-likeness and oral bioavailability criteria. MW, Molecular Weight; HBA, Hydrogen Bond Acceptors; HBD, Hydrogen Bond Donors; TPSA, Topological Polar Surface Area; WLogP, Water Partition Coefficient; RTB, Rotatable Bonds; CR, Coptidis Rhizoma; GR, Ginseng Radix; GRR, Glycyrrhizae Radix et Rhizoma; PT, Pinelliae Tuber; SR, Scutellariae Radix; ZF, Zizyphi Fructus; ZR, Zingiberis Rhizoma; ZRR, Zingiberis Rhizoma Recens.

The objective of this study was to investigate the human intestinal absorption of the individual compounds in BHSST using the log P method (WLogP), based on the BOILED-Egg model developed by Wildman and Crippen (Figure 1). This approach assesses two physicochemical properties: the topological polar surface area (TPSA) and lipophilicity [30]. The list of filtered compounds was refined to ensure suitability for further pharmacological analyses. After excluding compounds with predicted human oral bioavailability below 20%, and those that violated Lipinski’s “Rule of Five” or were P-glycoprotein substrates, we identified 23 out of 128 compounds from CR, 131 out of 716 compounds from GR, 285 out of 599 compounds from GRR, 78 out of 169 compounds from PT, 70 out of 232 compounds from SR, 181 out of 380 compounds from ZF, 281 out of 566 compounds from ZR, and 176 out of 442 compounds from ZRR. After removing duplicates, 982 compounds were identified as active compounds. The light histogram represents the entire set of compounds present in the formulation. In contrast, the dark histogram illustrates the distribution of the physicochemical properties of the 982 compounds that met the established criteria of high drug similarity and oral bioavailability (Figure 2). A comprehensive list of the active compounds is provided in Supplementary Material Table S1.

### 2.2. Candidate Target Proteins 

**Figure 3:**
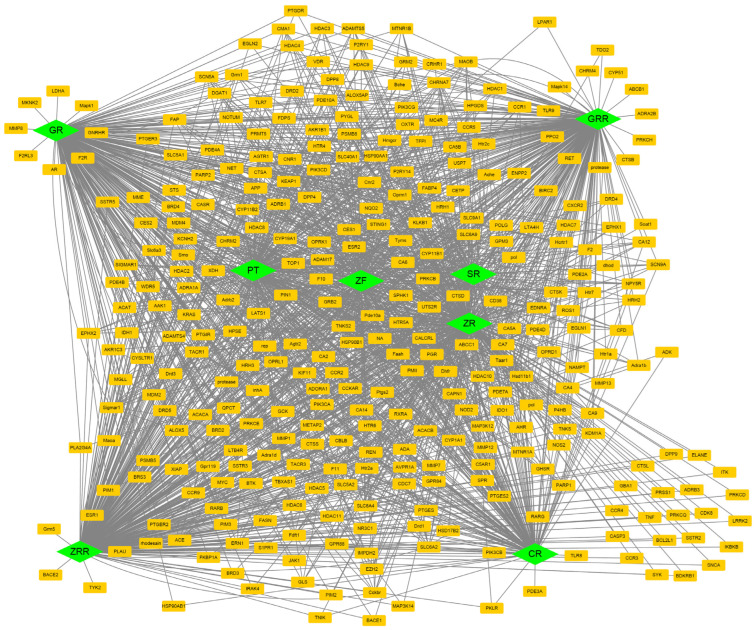
Network of components and target proteins. Green diamonds represent the components, while orange rectangles denote the target proteins. CR, Coptidis Rhizoma; GR, Ginseng Radix; GRR, Glycyrrhizae Radix et Rhizoma; PT, Pinelliae Tuber; SR, Scutellariae Radix; ZF, Zizyphi Fructus; ZR, Zingiberis Rhizoma; ZRR, Zingiberis Rhizoma Recens.

Version 33 of the ChEMBL database was used to predict candidate target proteins for each compound. The SMILES of the compounds were queried, and targets with a probability of 0.7 or higher were classified. After removing duplicates and retaining those relevant to Homo sapiens, 328 candidate targets for BHSST were identified (Figure 3; Supplementary Material Table S1). Supplementary Material Table S1 also shows the composition of the compound–target protein pairs.

### 2.3. Disease Gene Association

**Figure 4:**
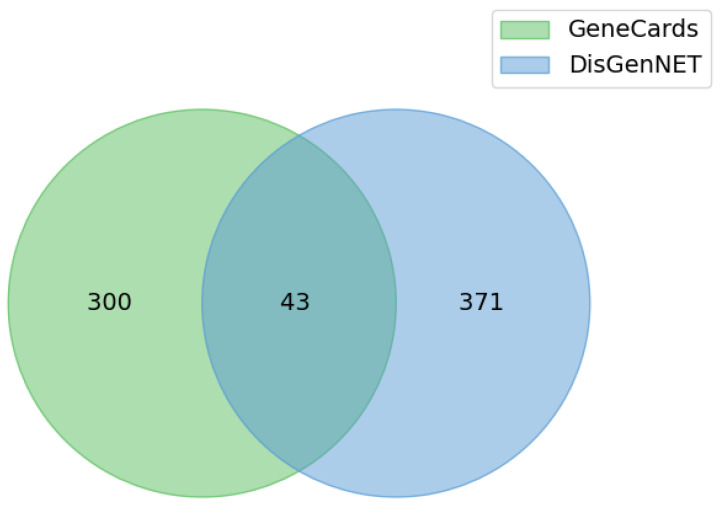
Venn diagram illustrating the overlap of IBS-related targets. Targets were selected on the basis of the top 10% of protein-coding genes from GeneCards and on the basis of targets with an evidence score of 0.7 or higher from DisGeNET.

To identify disease-related genes, we searched for the term “Irritable Bowel Syndrome” in the DisGeNET and GeneCards databases. This process yielded 414 IBS-related genes from DisGeNET, filtered for an evidence index of 0.7 or higher. Additionally, we identified 343 IBS-related protein-coding genes from the top 10% of the scores in the GeneCards database. Consequently, 43 overlapping targets were selected for further analysis (Figure 4).

### 2.4. Network Analysis

**Figure 5:**
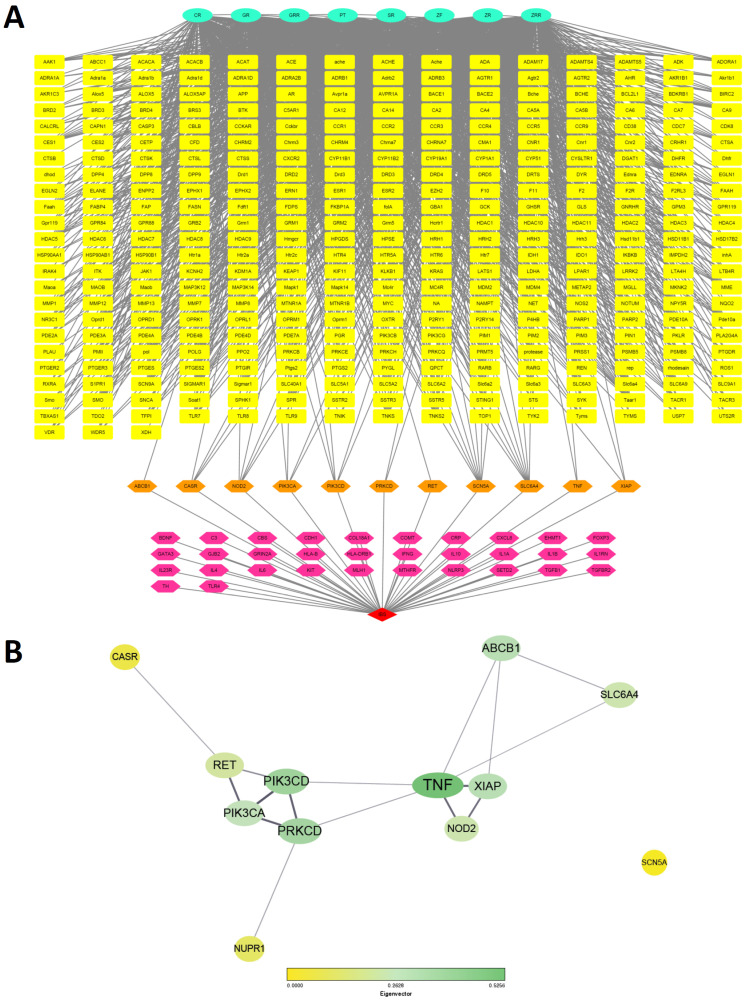
(**A**) Component–target–disease network for BHSST and IBS. Mint circles, yellow squares, pink hexagons, and red diamonds represent the components of BHSST, targets of BHSST, IBS-related targets, and IBS, respectively. Orange hexagons indicate potential BHSST targets involved in IBS pathogenesis. (**B**) Protein–protein interactions of 12 candidate target proteins from BHSST involved in IBS, as shown in panel (**A**). Node size and color represent degree and eigenvector centrality. CR: Coptidis Rhizoma, GR: Ginseng Radix, GRR: Glycyrrhizae Radix et Rhizoma, PT: Pinelliae Tuber, SR: Scutellariae Radix, ZF: Zizyphi Fructus, ZR: Zingiberis Rhizoma, ZRR: Zingiberis Rhizoma Recens.

The CTD network connecting BHSST with IBS was constructed using Cytoscape software and data from the STRING database (Figure 5A). Network centrality was assessed using the CytoNCA plug-in of Cytoscape (Table 1) [31]. The overlapping protein–protein interaction network between BHSST and IBS comprised 12 nodes and 16 edges (Figure 5B). In terms of degree centrality, TNF-α exhibited the highest centrality with a score of six, followed by PRKCD and PIK3CD. Regarding the eigenvector centrality, which reflects the centrality weights of neighboring nodes, TNF-α, PIK3CD, and PRKCD displayed relatively high eigenvector centrality, followed by XIAP and ABCB1. Specifically, we filtered key target proteins based on the 75th percentile of degree and eigenvector centrality. The third-quartile values were 4 for degree centrality and approximately 0.378 for eigenvector centrality. Accordingly, TNF-α (degree centrality: 6, eigenvector centrality: 0.526), PIK3CD (degree centrality: 4, eigenvector centrality: 0.394), and PRKCD (degree centrality: 4, eigenvector centrality: 0.371) were recognized as key targets (Table 1).

### 2.5. Enrichment Analysis of GO and KEGG Pathways

**Figure 6:**
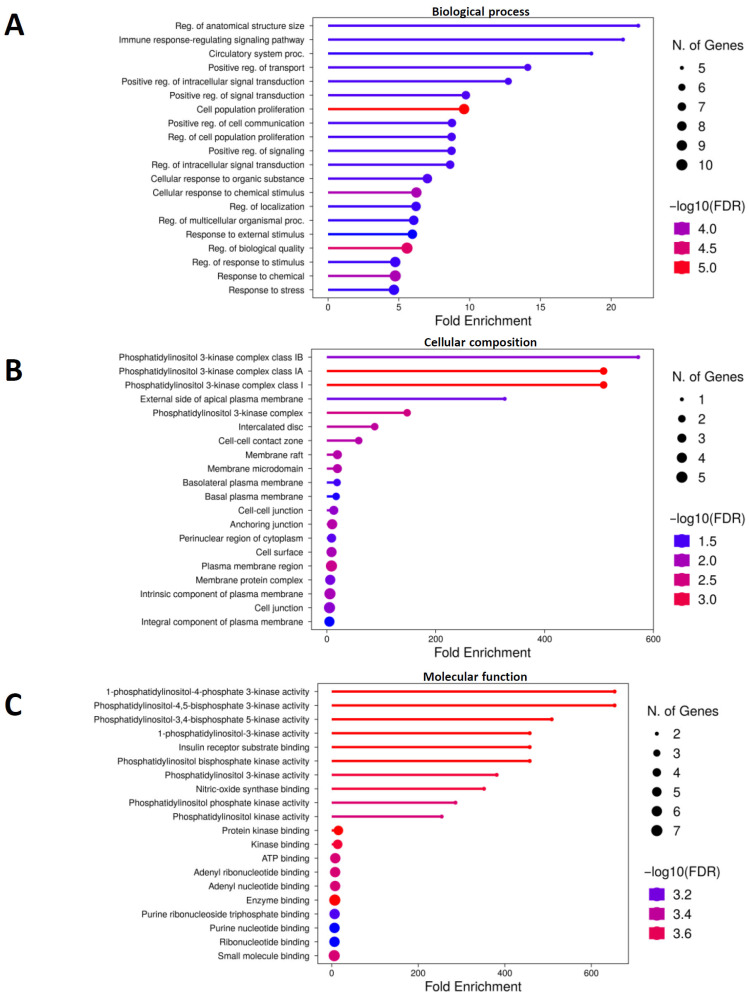
Gene ontology enrichment analysis. (**A**) Biological process, (**B**) cellular composition, and (**C**) molecular function. Based on the fold enrichment, the data are sorted in descending order. Bubble size represents the number of genes associated with each term (FDR cutoff < 0.05).

**Figure 7:**
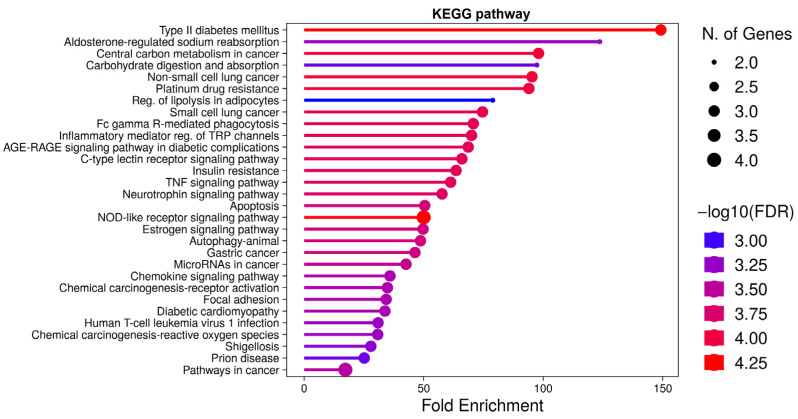
Enrichment analysis of the top 30 pathways from the Kyoto Encyclopedia of Genes and Genomes (KEGG). Based on fold enrichment, data are sorted in descending order. Bubble size represents the number of genes associated with each term (FDR cutoff < 0.05).

GO enrichment analysis was conducted to explore the biological processes related to the 12 overlapping target candidates of BHSST implicated in IBS. The GO analysis results were categorized according to high-fold enrichment and low false discovery rates. The results indicated enrichment in the following biological processes: regulation of anatomical structure size, immune response-regulating signaling pathways, circulatory system processes, positive regulation of transport, and positive regulation of intracellular signal transduction. The predicted candidate locations for cellular components included the phosphatidylinositol 3-kinase complex class IA, phosphatidylinositol 3-kinase complex class IB, phosphatidylinositol 3-kinase complex class I, the external side of the apical plasma membrane, and the phosphatidylinositol 3-kinase complex. The following activities were enriched: phosphatidylinositol-4,5-bisphosphate 3-kinase activity; phosphatidylinositol-3,4-bisphosphate 5-kinase activity; 1-phosphatidylinositol-4-phosphate 3-kinase; 1-phosphatidylinositol-3-kinase; and insulin receptor substrate binding (Figure 6). To further elucidate the potential mechanisms, we investigated enriched biological pathways using the KEGG database. The results showed significant enrichment of pathways related to type II diabetes mellitus, central carbon metabolism in cancer, aldosterone-regulated sodium reabsorption, carbohydrate digestion and absorption, non-small-cell lung cancer, and platinum drug resistance (Figure 7). Excluding diseases unrelated to IBS, the TNF-α signaling pathway was notably enriched. Overall, TNF-α signaling-related proteins are potential candidates for the regulatory effects of BHSST on IBS.

### 2.6. Molecular Docking Simulation

**Figure 8:**
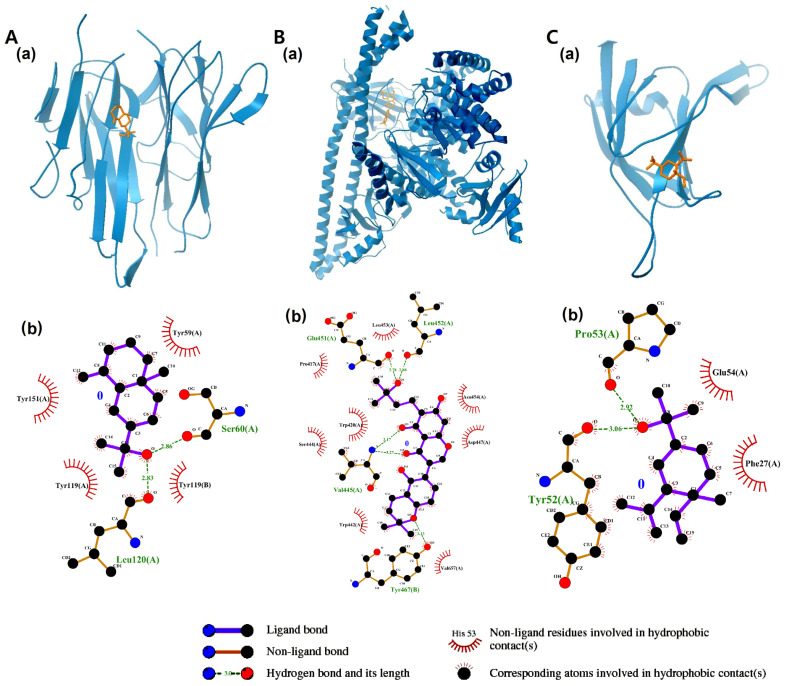
Representative stable binding poses from molecular docking simulations. The top panel shows the docking poses of the ligand (orange) with the protein (blue cartoon). The bottom panel illustrates the molecular interactions between the ligand and the protein, indicating the residues and chains involved in the interactions. Hydrogen bonds are depicted as green dashed lines with distances (in Ångströms). (**A**) Eudem-4(14)-en-11-ol with TNF-α. (**B**) Kanzonol T with PIK3CD. (**C**) Elemol with PRKCD. Black beads represent carbon, red beads represent oxygen, and blue beads represent nitrogen.

Based on the network and enrichment analyses that identified TNF-α, PIK3CD, and PRKCD as key targets, molecular docking simulations were performed to determine whether the active compounds of BHSST could stably bind to these core proteins. Ligand-binding pockets of each protein were identified using the Protein Data Bank and literature sources. Ten independent molecular docking simulations were conducted for active compounds targeting TNF-α, PIK3CD, and PRKCD. Most of the simulation results showed stable binding positions consistent with those of known inhibitors. Binding poses with binding affinities lower than −5 kcal/mol are generally considered stable. Molecular docking simulations showed binding poses with affinities primarily below −5 kcal/mol (Supplementary Material Table S2). Specifically, the lowest binding affinity for TNF-α (PDB ID: 5MU8) with eudesm-4(14)-en-11-ol from ZR was −6.8212 kcal/mol, that for PIK3CD (PDB ID: 6PYR) with kanzonol T from GRR was −8.6546 kcal/mol, and that for PRKCD (PDB ID: 1YRK) with elemol from ZR and ZRR was −5.8467 kcal/mol (Figure 8, Table 2). A list of other candidate binding positions is provided in Supplementary Material Table S2.

### 2.7. Molecular Dynamics Results

**Figure 9:**
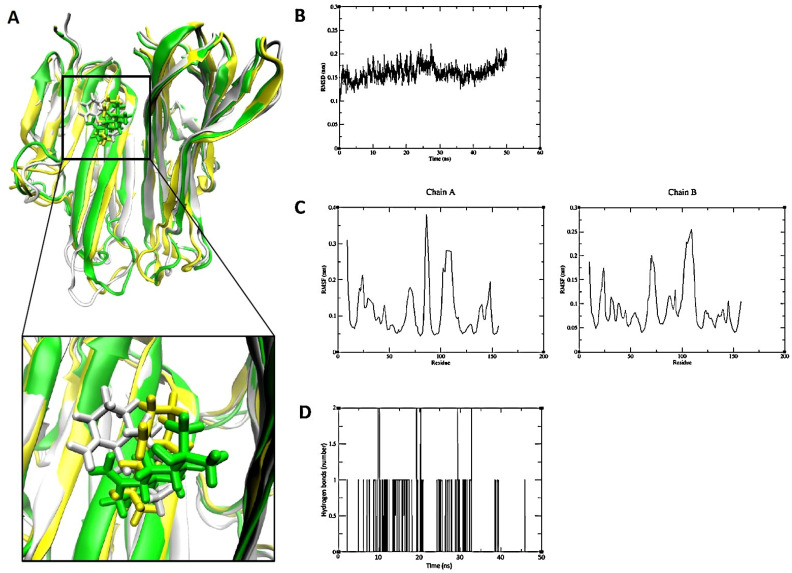
Molecular dynamics simulation analysis of eudem-4(14)-en-11-ol binding to the TNF-α dimer. (**A**) Three conformational snapshots merged at 25 ns intervals during the simulation (white: 0 ns; yellow: 25 ns; green: 50 ns). (**B**) RMSD between eudem-4(14)-en-11-ol and TNF-α. (**C**) RMSF of TNF-α. (**D**) Hydrogen bond interactions between eudem-4(14)-en-11-ol and TNF-α.

To corroborate the results obtained from the molecular docking analysis, a series of molecular dynamics (MD) simulations were conducted using GROMACS software in conjunction with the Charmm36 force field. The simulation results for TNF-α indicated that eudem-4(14)-en-11-ol stably bound to the protein over 50 ns. The RMSD of the distance between eu-dem-4(14)-en-11-ol and TNF-α was 0.16 nm, with a standard deviation of 0.02 nm. To gain insight into the trajectory of the ligand and protein, three data points were obtained at 25 ns intervals. Eudem-4(14)-en-11-ol remained stable within the cavity between the TNF-α dimers throughout the simulation. It was further demonstrated that the consistently maintained hydrogen bond interactions provided additional support for the binding of eu-dem-4(14)-en-11-ol to TNF-α (Figure 9).

**Figure 10:**
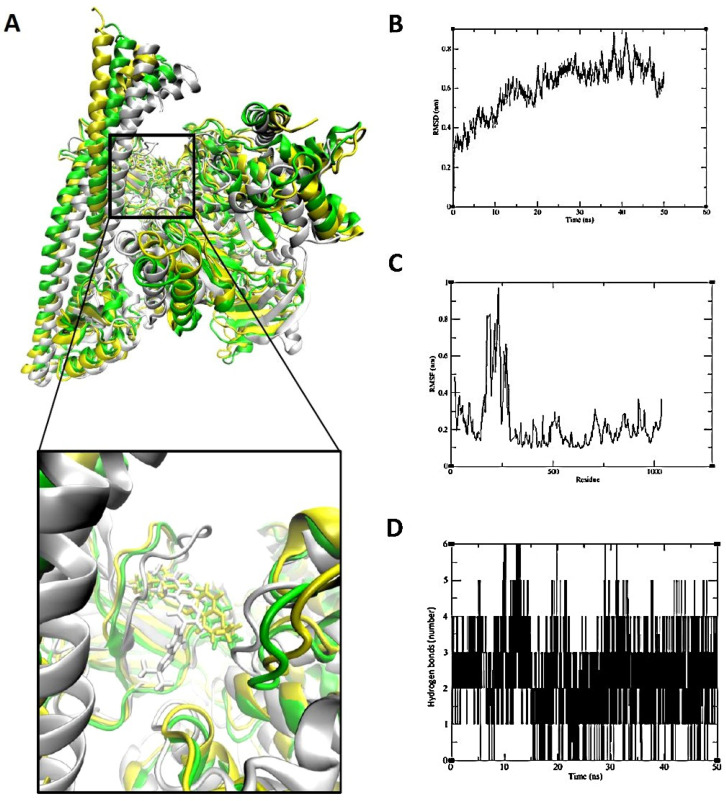
Analysis of the binding of Kanzonol T to PIK3CD by molecular dynamics simulation. (**A**) Three conformational snapshots taken at 25 ns intervals during the simulation (white: 0 ns; yellow: 25 ns; green: 50 ns). The molecular surface of PIK3CD is shown in white at 50 ns. (**B**) RMSD between Kanzonol T and PIK3CD. (**C**) RMSF of PIK3CD. (**D**) Interactions of the hydrogen bonds between Kanzonol T and PIK3CD.

The binding of kanzonol T to PIK3CD was maintained at the active site. Although the planar conformation folded quickly, the portion of the structure bound near the active site pocket remained stable throughout the simulation. The RMSD of the distance was calculated to be 0.6 nm, with a standard deviation of 0.14 nm. Hydrogen bonding interactions remained consistently stable (Figure 10).

**Figure 11:**
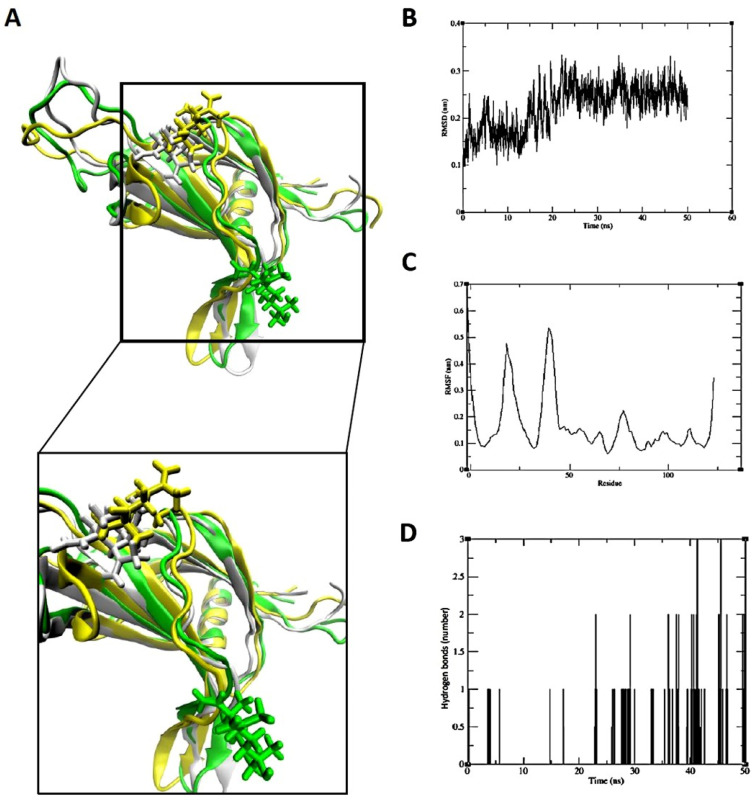
Analysis of the binding of elemol binding to PRKCD by molecular dynamics simulation. (**A**) Four conformational snapshots taken during the simulation and merged (white: 0 ns; yellow: 25 ns; green: 50 ns). Elemol moved away from the initial binding site on the catalytic domain over the course of the simulation. The molecular surface of PRKCD is shown in white at 50 ns. (**B**) RMSD between elemol and PRKCD. (**C**) RMSF of PRKCD. (**D**) Interactions of the hydrogen bonds between elemol and PRKCD.

However, MD simulation of elemol with PRKCD yielded results that differed from those obtained by molecular docking. The average RMSD was 0.28 nm, with a standard deviation of 0.11 nm. Elemol maintained its binding position from the start of the simulation until approximately 25 ns; however, at 50 ns it deviated slightly from the catalytic region (Figure 11).

### 2.8. Effects of Eudesm-4(14)-en-11-ol, Elemol, and BHSST on Zymosan-Induced Colon Alterations in IBS Mouse Models

**Figure 12:**
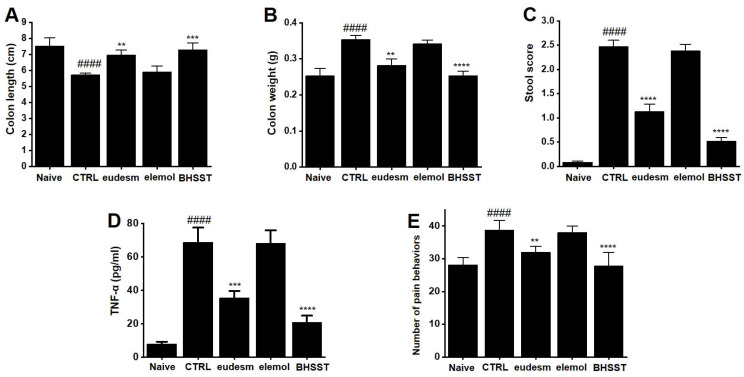
Effects of eudesm-4(14)-en-11-ol, elemol, and BHSST on colonic characteristics, stool consistency, TNF-α mRNA expression, and nociceptive behaviors in a zymosan-induced IBS animal model. (**A**) Colonic length and (**B**) weight were measured. (**C**) Stool consistency was assessed using a scoring system. (**D**) TNF-α mRNA levels were quantified by RT-qPCR. (E) Visceral pain-related behaviors were examined. Data are presented as mean ± SE. #### *p* < 0.0001 compared to the naïve group; ** *p* < 0.01, *** *p* < 0.001 and **** *p* < 0.0001 compared to the control group. CTRL: Control group; Eudesm: eudesm-4(14)-en-11-ol; BHSST: Banhasasim-tang.

To evaluate the therapeutic potential of eudesm-4(14)-en-11-ol, elemol, and BHSST, we assessed colon length and weight, stool characteristics, TNF-α levels, and pain-related behaviors in a zymosan-induced IBS colitis mouse model. Zymosan administration significantly shortened colon length and increased colon weight compared to healthy controls, confirming the successful induction of colitis. Treatment with eudesm-4(14)-en-11-ol (100 mg/kg) significantly restored colon length and reduced colon weight, while elemol (100 mg/kg) showed no measurable effect. BHSST (500 mg/kg) produced outcomes comparable to those of eudesm-4(14)-en-11-ol (Figure 12A,B). Zymosan also induced stool abnormalities, including watery, discolored feces and increased stool consistency scores. These alterations were significantly ameliorated by both eudesm-4(14)-en-11-ol and BHSST but remained unchanged with elemol treatment (Figure 12C). Furthermore, eudesm-4(14)-en-11-ol and BHSST both significantly reduced TNF-α expression and attenuated pain-related behaviors, while elemol did not demonstrate any beneficial effects (Figure 12D,E). Collectively, these findings suggest that eudesm-4(14)-en-11-ol, a bioactive component of BHSST, contributes substantially to the alleviation of zymosan-induced colitis and its associated symptoms.

## 3. Discussion

This study employed network pharmacology to explore the potential therapeutic targets and pathways of BHSST in the context of IBS. Prior to conducting the analysis, it was necessary to identify the physicochemical properties of the individual compounds present in each herbal component of BHSST, which required access to comprehensive metadata. Several specialized databases provide systematically curated information on traditional medicines, including the TM-MC, Zhongyaocai Integrated Database, TCM Database@Taiwan, and the Traditional Chinese Medicine Systems Pharmacology Database and Analysis Platform [32,33,34,35]. Among these, TM-MC was selected as the primary resource for the following three main reasons: (1) it focuses on traditional medicines commonly used in Northeast Asia; (2) the database is curated by subject-matter experts, including biologists and traditional medicine practitioners, based on up-to-date literature; (3) it offers clearly referenced and well-structured data sources.

To screen candidate therapeutic compounds, two criteria were applied: drug-likeness and oral bioavailability. While the definition of drug-likeness may vary across studies, compounds with higher drug-likeness scores are generally considered more likely to exhibit therapeutic effects. Oral bioavailability refers to the proportion of an orally administered compound that reaches systemic circulation and is thus available for pharmacological action [36].

Lipinski reported that compounds with poor absorption and permeability are more likely to fail in early-phase clinical trials and, thus, are considered to have low drug-likeness [37]. To address this, he proposed the well-known “Rule of 5”, which outlines four criteria for optimal drug-likeness: a molecular weight under 500 Da, no more than five hydrogen bond donors, no more than ten hydrogen bond acceptors, and a calculated LogP below 5 [38]. Although several approved drugs, including antibiotics and antifungals, violate this rule, it remains a widely used guideline for predicting oral bioavailability. Similarly, Veber suggested that favorable oral bioavailability is associated with specific molecular properties, such as fewer than 10 rotatable bonds and a topological polar surface area (TPSA) below 140 Å^2^ [39]. Based on these principles, the BOILED-Egg model was applied in our study to predict human intestinal absorption and blood–brain barrier (BBB) permeability. This model, developed from experimental datasets [40], utilizes two key physicochemical parameters—TPSA and WLogP—to classify compounds. The supervised learning algorithm used in the BOILED-Egg model demonstrated reliable predictive power, with a Matthews correlation coefficient of 0.6531 and an accuracy of 0.9156 for intestinal absorption [30]. Compounds predicted to be poorly absorbed were excluded from further analysis. Additionally, compounds identified as P-glycoprotein substrates were removed as P-glycoprotein plays a critical role in effluxing xenobiotics and their metabolites, thereby limiting therapeutic efficacy [41]. Oral bioavailability was further evaluated using Wei’s model, applying a 20% cutoff threshold [37]. This model, trained on 1142 molecules and validated with 287 test molecules, showed robust predictive performance. Interestingly, among over 1000 molecular descriptors, topological indices exhibited a negative correlation with oral bioavailability [37,42].

After removing duplicates, 328 compounds were identified as active constituents of BHSST. Given the limitations of rule-based drug screening approaches, we combined both rule-based criteria and machine learning models to select candidate compounds predicted to have favorable drug-likeness and oral bioavailability in silico. Figure 2 illustrates the histograms of the physicochemical properties of BHSST compounds: active compounds (black) versus all compounds (white). The observed distributional differences underscore the insufficiency of rule-based methods alone in accurately identifying bioactive compounds. Subsequently, IBS-related genes were retrieved from the GeneCards and DisGeNET databases. From GeneCards the top 10% of genes ranked by relevance score were selected, while from DisGeNET genes with an evidence index ≥ 0.7 were included. The overlapping genes were subjected to further analysis.

To examine the potential interaction between BHSST and IBS-related targets, a compound–target–disease (CTD) network was constructed. The intersection of compound-derived and disease-related targets suggested mechanistic links through which BHSST might exert therapeutic effects in IBS. From this network, 12 hub targets (pivot candidates) were identified, and their protein–protein interaction (PPI) networks were analyzed. Such topological network analysis is known to provide novel insights into biological complexity and supports drug repurposing efforts [43].

Centrality measures, commonly applied in topological analyses, help to assess the importance of individual nodes within a biological network. Degree centrality quantifies the number of direct interactions, while eigenvector centrality reflects node influence based on the connectivity of neighboring nodes [44]. Although betweenness and closeness centralities are also standard metrics, they require biological interpretation and were not the primary focus of this study. Instead, we emphasized degree and eigenvector centralities to assess node importance. Nonetheless, for reference, data on betweenness and closeness centrality are also provided (Table 1).

Degree and eigenvector centrality analyses identified TNF-α, PIK3CD, and PRKCD as key regulatory nodes in IBS pathophysiology. TNF-α is a pivotal mediator of inflammation, and the monoclonal antibody infliximab, which targets TNF-α, is widely used to treat inflammatory diseases such as Crohn’s disease and ulcerative colitis [45]. Anti-TNF-α therapies not only reduce inflammation but also help restore gut microbiota composition toward a healthier state in patients with Crohn’s disease [46]. Given increasing evidence that low-grade inflammation and dysbiosis contribute to IBS symptoms, modulation of TNF-α may offer therapeutic benefits in specific IBS subtypes, especially those characterized by immune activation or post-infectious features. PIK3CD plays a critical role in immune cell signaling [47]. Gain-of-function mutations in PIK3CD have been linked to immunodeficiency syndromes presenting with gastrointestinal symptoms, including chronic diarrhea, intestinal lymphoid hyperplasia, and mucosal inflammation [48]. These manifestations overlap with clinical features observed in inflammatory IBS subtypes. Therefore, targeting PIK3CD signaling may represent a promising approach to modulate aberrant immune responses and restore gut homeostasis in IBS patients with underlying immune dysregulation. PRKCD, a member of the protein kinase C family, regulates diverse cellular functions such as apoptosis, proliferation, and immune cell activity [49]. Within the gut, PRKCD may influence epithelial barrier integrity and immune tolerance through its context-dependent pro- or anti-apoptotic effects [50]. Dysregulation of PRKCD signaling could contribute to mucosal immune imbalance, visceral hypersensitivity, or impaired epithelial repair in IBS. Thus, modulating PRKCD activity may have therapeutic relevance, although further studies are necessary to elucidate its precise role in IBS pathogenesis.

Molecular docking is a computational technique used to predict the preferred binding positions of ligands to target proteins. In this study, docking simulations revealed stable binding affinities between active compounds from BHSST and the key target proteins TNF-α, PIK3CD, and PRKCD. Previous research suggests that binding affinities around −5 kcal/mol indicate stable ligand–protein interactions [51,52]. Consistently, several active compounds in our study exhibited binding affinities of −5 kcal/mol or lower, implying potential effective interactions with these targets (see Supplementary Material Table S2). MD simulations, based on molecular mechanics, provide a dynamic view by tracking the trajectories of molecules over time [53]. While molecular docking offers a high-throughput assessment of ligand–receptor interactions, it represents a static snapshot and cannot capture the full dynamic behavior of molecular complexes. Therefore, we complemented docking results with MD simulations to further validate and characterize the stability and dynamics of the ligand–target interactions.

However, this study has several limitations. First, although Lipinski’s rule of five was used to screen drug-like compounds, it may not be fully applicable to complex herbal formulations like BHSST, which contain a broad range of phytochemicals that do not conform to typical synthetic drug properties. Second, the oral bioavailability and systemic exposure of the predicted active compounds, including eudesm-4(14)-en-11-ol, have not been evaluated in humans. Therefore, their actual therapeutic potential in clinical settings remains to be validated. Also, while molecular docking and dynamics analyses suggest plausible target interactions, these findings should be supported by further pharmacokinetic and pharmacodynamic studies. In addition, although in silico approaches such as molecular docking and dynamics simulations provided valuable insights into potential compound–target interactions, these computational findings ideally require further validation through in vitro assays, including enzymatic inhibition tests or biophysical binding analyses such as surface plasmon resonance, isothermal titration calorimetry, or microscale thermophoresis. Due to current laboratory limitations, we were unable to perform such in vitro validation in this study. Instead, we focused on in vivo experiments to partially support the predicted mechanisms. Nevertheless, future studies incorporating these functional assays will be essential to confirm the direct molecular interactions and further substantiate the pharmacological relevance of the identified compounds. Furthermore, the extract used in this study was provided as a finished product by a pharmaceutical company, and critical manufacturing details such as the drug extract ratio (DER) and precise phytochemical composition were not disclosed. This lack of quantitative characterization may affect the consistency and reproducibility of pharmacological effects across different batches. Future studies should aim to include comprehensive phytochemical profiling and batch standardization to ensure product quality and replicable biological activity. Moreover, while selected compounds such as eudesm-4(14)-en-11-ol and elemol were administered in animal experiments based on available literature, their actual content within the BHSST extract was not quantified. This limits our ability to directly correlate their concentrations with observed pharmacological outcomes. Inclusion of validated analytical methods to determine the levels of such constituents would enhance the reliability and reproducibility of future research.

Consequently, eudesm-4(14)-en-11-ol was found to bind stably within the central cavity of the TNF-α dimer (Figure 9). The stable root mean square deviation (RMSD) and trajectory analysis indicate a tight and sustained interaction between eudesm-4(14)-en-11-ol and TNF-α. The substrate-binding pocket of PIK3CD, comprising residues Trp442, Leu452, and Tyr467, mediates interactions with various substrates and inhibitors primarily through hydrogen bonding. Molecular dynamics simulations demonstrated that kanzonol T maintained a stable binding conformation within this pocket (Figure 10). Interestingly, the MD analysis of elemol binding to PRKCD revealed results that differed from the initial molecular docking predictions. Although docking simulations suggested a stable binding pose with an affinity of −5.8467 kcal/mol, MD simulations showed that elemol deviated from its initial binding position after approximately 50 ns (Figure 11), indicating reduced stability in a dynamic environment.

Among the molecular candidates identified, eudesm-4(14)-en-11-ol and kanzonol T were prioritized for further evaluation due to their predicted biological activity. However, owing to the unavailability of commercially sourced kanzonol T, we were unable to include it in the in vivo experiments and thus could only confirm the therapeutic efficacy of eudesm-4(14)-en-11-ol in the IBS model. As a complementary control, elemol was also evaluated and showed no therapeutic effect. Notably, BHSST treatment produced beneficial effects comparable to those of eudesm-4(14)-en-11-ol (Figure 12). Future studies will focus on the isolation or synthesis of kanzonol T to enable its biological evaluation in similar in vivo models.

## 4. Materials and Methods

### 4.1. Screening Active Compounds

The list of compounds in BHSST was retrieved from the Traditional Medicine Materials and Chemical Compounds Database of Northeast Asia (https://informatics.kiom.re.kr/compound/, accessed on 2 May 2024) [32]. The Simplified Molecular Input Line Entry System (SMILES) for each compound was retrieved and validated using the PubChem database (https://pubchem.ncbi.nlm.nih.gov/, accessed on 2 May 2024). If the isomeric SMILES of a compound was not available in the PubChem database, the standard SMILES was selected for analysis. Compounds predicted to be negative for P-glycoprotein substrates and to exhibit human intestinal absorption were classified using SwissADME (http://www.swissadme.ch/index.php/, accessed on 7 May 2024) based on SMILES representations of up to 200 characters. The remaining compounds, which did not violate Lipinski’s “Rule of Five” and were predicted to have an oral bioavailability exceeding 20%, were identified as active compounds [37,38].

### 4.2. Selection of Disease-Related Genes

IBS-related genes were retrieved from GeneCards (https://www.genecards.org/, accessed on 7 May 2024) and DisGeNET (https://www.disgenet.org/, accessed on 7 May 2024) databases [54,55]. To maintain a high level of relevance between diseases and genes, the top 10% protein-coding genes from GeneCards and those with an evidence index of 0.7 or above from DisGeNET were selected.

### 4.3. Target Prediction

The target proteins of the active compounds in BHSST were predicted using models from version 33 of the ChEMBL database (https://www.ebi.ac.uk/chembl/, accessed on 13 May 2024) [56,57]. The SMILES of each compound were queried using the ChEMBL multitask neural network model (https://github.com/chembl/chembl_multitask_model accessed on 13 May 2024), and the target proteins were filtered for those with a probability of 0.7 or higher for human targets.

### 4.4. Network Construction

To elucidate the associations among active compounds, target candidates, and diseases, a CTD network was constructed. Key protein–protein interactions were obtained using Cytoscape 3.9.1 software (Boston, MA, USA) [56]. Critical nodes in the protein–protein interactions were identified using the third quartile of eigenvector centrality and degree centrality.

### 4.5. Gene Ontology (GO) and Kyoto Encyclopedia of Genes and Genomes (KEGG) Pathway Analysis

To elucidate the contributions of BHSST to the functional and underlying pathways, GO and KEGG pathways were analyzed [57,58]. GO and KEGG pathway analyses were conducted using ShinyGO version 0.80 (http://bioinformatics.sdstate.edu/go/, accessed on 29 May 2024) [59]. A statistical cutoff threshold of 0.05 was established. The top 20 GO results and the top 30 KEGG pathway results were reported based on fold enrichment, which reflected the false discovery rate and gene count.

### 4.6. Molecular Docking

The crystal structures of the target proteins were sourced from the RCSB Protein Data Bank (https://www.rcsb.org/, accessed on 11 June 2024), and the three-dimensional structures of each ligand were prepared using the PubChem database. Prior to the docking simulations, heteroatoms, including ions and water molecules, were removed. The ligand files were converted into AutoDock-compatible formats (.sdf to .pdb) using PyMOL software (V3.0.5; Schrödinger LLC., New York, NY, USA). Ligands were prepared to identify rotatable bonds, charges, and unnecessary hydrogen atoms. Proteins were processed to adjust charges, add hydrogen atoms, and merge non-polar hydrogen atoms for docking. A binding site search grid was configured to cover the entire surface of the protein. To ensure accuracy, ten independent docking simulations were conducted for each ligand, with the simulation completeness set to 32. The docking simulations were performed using AutoDock Tools version 1.5.7 and AutoDock Vina version 1.2.3 [60,61,62]. The pose exhibiting the lowest binding affinity (kcal/mol) between the active compounds and proteins was considered the most stable protein–ligand binding pose. Data visualization was performed using PyMOL version 2.6 and LigPlot+ version 2.2 software [63].

### 4.7. Molecular Dynamics Simulation

GROMACS software version 2024.2 was used for the molecular dynamics (MD) simulations [64]. Prior to initiating the simulations, each protein structure was subjected to a preparation process wherein water molecules and heteroatoms were removed from the coordinate files in accordance with established protocols. The coordinates of the active compounds were selected based on the results of molecular docking simulations, which identified the most stable binding poses. The coordinates and parameters of each active compound were configured according to the CHARMM36 force field in the context of MD [65]. In order to model the solvation under periodic boundary conditions, the TIP3P explicit water model was employed. The objective of this study was to introduce sodium and chloride ions at a concentration of 0.154 M to achieve physiological conditions. Additional sodium ions were added to neutralize the system. The system was subsequently subjected to energy minimization. For NVT and NPT equilibrations, the Berendsen thermostat and Parrinello–Rahman barostat, respectively, were utilized. In this study, long-range electrostatics were investigated using the Particle Mesh Ewald method [66]. A neighbor-searching operation was conducted using the Verlet algorithm. Following a 50 ns simulation, the root mean square deviation (RMSD) and root mean square fluctuation (RMSF) were analyzed. The hydrogen bonding interactions during the simulation were evaluated using the VMD software (V1.9.4.) [67], employing distance and angle cutoffs of 3.5 nm and 30°, respectively. The results were rendered in a three-dimensional visual form using PyMOL software.

### 4.8. Instrument and Reagents

BHSST was donated by HANKOOKSHINYAK Pharmaceutical Co., Ltd. (Nonsan, Republic of Korea). The dried extract powder of BHSST, which was prepared using a water extraction method, was used in the present study. The analysis was conducted using an ACQUITY™ UPLC system (Waters, Milford, MA, USA), equipped with a photodiode array (PDA) detector and an ACQUITY™ BEH C18 column (1.7 μm, 2.1 × 100 mm). Data acquisition and processing were carried out using Empower software (V3.8.0). For sample extraction, a microwave extractor (Power Sonic 405, HWA-SHIN TECH, Seoul, Republic of Korea) was employed. The solvents used included methanol (Junsei, Tokyo, Japan), acetonitrile (JT BAKER, Phillipsburg, NJ, USA), and triple-distilled water. The individual initial components of BHSST are presented in Table 3. Reference standards—6-gingerol, glycyrrhizinic acid, liquiritin, isoliquiritigenin, berberine, baicalin, and ginsenoside Rb1—were obtained from Sigma-Aldrich (St. Louis, MO, USA) [27,68].

### 4.9. Preparation of Standard Solutions

For quantitative analysis, precise amounts of each reference compound—6-gingerol, glycyrrhizinic acid, liquiritin, isoliquiritigenin, berberine, baicalin, and ginsenoside Rb1—were accurately weighed and dissolved in methanol to prepare stock solutions at a concentration of 1 μg/mL. These stock solutions were subsequently diluted with methanol to obtain working standard solutions at concentrations of 1, 5, and 10 ng/mL [27,68].

### 4.10. Preparation of Test Solution for Quantitative Analysis

For the quantitative analysis, each test solution was prepared by accurately weighing 0.1 g of the BHSST sample, which had been uniformly mixed with the respective test liquid. The weighed sample was then extracted with 10 mL of 50% methanol using microwave-assisted extraction for 1 h. After extraction, the mixture was filtered through a membrane filter with a pore size of less than 0.45 μm, and the resulting filtrate was collected for analysis [27,68].

### 4.11. Quantification of BHSST

Quantitative analysis of BHSST was performed using a Waters ACQUITY™ UPLC system equipped with a photodiode array (PDA) detector and an ACQUITY™ BEH C18 column (1.7 μm, 2.1 × 100 mm, Waters, Milford, MA, USA). Data acquisition was managed using Empower software. The column was maintained at ambient temperature. The PDA detection wavelengths for the analytes were as follows: 6-gingerol at 280 nm; glycyrrhizinic acid, liquiritin, and isoliquiritigenin at 254 nm; berberine at 345 nm; and baicalin at 277 nm. The mobile phase consisted of acetonitrile and water containing 0.1% formic acid. Detailed chromatographic conditions are provided in Table 4. Each sample (10 μL) was injected with a flow rate of 1.0 mL/min. Identification was based on retention times, and quantification was carried out using the peak area method.

For the analysis of ginsenoside Rg1, PDA detection was performed at 203 nm. The mobile phase consisted of a binary mixture of acetonitrile and water. Analytical parameters specific to Rg1 are summarized in Table 5. In this case, a 2 μL sample was injected with a flow rate of 0.4 mL/min. As with the other analytes, identification was determined by retention time, and quantification was based on peak area [27,68].

### 4.12. Quantitative Analysis of Marker Compounds in BHSST

The concentrations of 6-gingerol, glycyrrhizinic acid, liquiritin, isoliquiritigenin, berberine, baicalin, and ginsenoside Rb1 in BHSST were determined by UPLC. Quantification was performed based on calibration curves generated from each corresponding reference standard, as shown in Table 6. Method validation confirmed the analytical procedure to be stable and reliable, enabling consistent and effective separation of the seven major constituents in BHSST [27,68].

### 4.13. IBS Animal Model and Treatment

Colitis was induced in 6-week-old C57BL/6 mice via transanal administration of 0.1 mL of a zymosan suspension (30 mg/mL) using a feeding needle. Mice were then orally administered one of the following treatments once daily for three consecutive days: PBS, eudesm-4(14)-en-11-ol (100 mg/kg; Sigma-Aldrich, St. Louis, MO, USA) [69], elemol (100 mg/kg; Angene Chemical, London, UK), or BHSST (500 mg/kg) [27,68]. On day four all experimental assessments were conducted. The zymosan-treated mice were divided into the following five groups (n = 8 per group): PBS naïve, zymosan control, eudesm-4(14)-en-11-ol-treated, elemol-treated, and BHSST-treated.

### 4.14. Macroscopic Evaluation

After fecal matter was removed, colon length (from cecum to anus) and weight were measured. Stool consistency was scored independently by three blinded observers using a four-point scale: 0 = normal, 1 = loose, 2 = soft/sticky, and 3 = diarrhea.

### 4.15. Quantification of TNF-α Expression by RT-qPCR

Total RNA was extracted from colonic tissue using TRIzol reagent (Invitrogen, Waltham, MA, USA). Complementary DNA (cDNA) was synthesized from the isolated RNA using an M-MLV reverse transcriptase kit (Promega, Madison, WI, USA). TNF-α mRNA expression levels were quantified using real-time quantitative PCR (RT-qPCR).

### 4.16. Assessment of Pain-Related Behaviors

Pain-related behaviors were evaluated by observing specific actions such as abdominal licking, full-body stretching, pressing the abdomen against the floor, and arching of the back caused by visceral contractions. Two independent observers recorded these behaviors during a 10 min observation period.

### 4.17. Statistical Analysis

Data are presented as mean ± standard error (SE). Statistical comparisons among groups were performed using a one-way analysis of variance (ANOVA), followed by Dunnett’s post hoc test. Analyses were conducted using GraphPad Prism 8 software, with statistical significance set at *p* < 0.05.

## 5. Conclusions

This study employed network pharmacology, molecular docking, and molecular dynamics simulations to explore the potential mechanisms of BHSST in IBS. In silico analyses identified 12 candidate targets, highlighting the TNF-α signaling and apoptosis pathways. Among the compounds analyzed, eudesm-4(14)-en-11-ol and kanzonol T showed potential interactions with TNF-α and PIK3CD, respectively. In vivo validation using a zymosan-induced IBS mouse model confirmed the therapeutic potential of eudesm-4(14)-en-11-ol, while elemol, predicted to be inactive, showed no effect. BHSST itself produced similar improvements to eudesm-4(14)-en-11-ol across key parameters. These findings offer preliminary mechanistic insights into the anti-inflammatory actions of BHSST and support its potential relevance in IBS management, warranting further investigation.

## Figures and Tables

**Table 1:** Centrality analysis of 12 candidate target proteins related to irritable bowel syndrome in Banhasasim-tang.

Protein Name	Gene Symbol	Degree	Eigenvector	Betweenness	Closeness
Tumor necrosis factor, membrane form	* TNF-α *	6	0.526	52	0.407
Phosphatidylinositol 4,5-bisphosphate 3-kinase catalytic subunit delta isoform	* PIK3CD *	4	0.394	29	0.393
Protein kinase C delta-type regulatory subunit	* PRKCD *	4	0.371	23	0.379
ATP-dependent translocase ABCB1	* ABCB1 *	3	0.304	1	0.324
E3 ubiquitin-protein ligase XIAP	* XIAP *	3	0.304	1	0.324
Phosphatidylinositol 4,5-bisphosphate 3-kinase catalytic subunit alpha isoform	* PIK3CA *	3	0.277	4	0.333
Sodium-dependent serotonin transporter	* SLC6A4 *	2	0.236	0	0.314
Nucleotide-binding oligomerization domain-containing protein 2	* NOD2 *	2	0.236	0	0.314
Extracellular cell-membrane anchored RET cadherin 120 kDa fragment	* RET *	3	0.208	18	0.324
Nuclear protein 1	* NUPR1 *	1	0.106	0	0.289
Extracellular calcium-sensing receptor	* CASR *	1	0.059	0	0.256
Sodium channel protein type 5 subunit alpha	* SCN5A *	0	0.000	0	0.083

**Table 2:** Representative molecular docking simulation results for active compounds in BHSST with their target proteins.

Medicine	Compound Name	PubChem ID	Target Protein (PDB ID)	Binding Affinity (kcal/mol)
ZR	eudesm-4(14)-en-11-ol	10557	TNF-α (5MU8)	−6.8212
GRR	kanzonol T	999902	PIK3CD (6PYR)	−8.6546
ZR, ZRR	elemol	92138	PRKCD (1YRK)	−5.8467

**Table 3:** Initial composition of BHSST used in this study.

Latin Name	Scientific Name	Amount (g)
Pinelliae rhizoma	*Pinellia ternata* Breitenbach	0.63
Scutellariae radix	*Scutellaria baicalensis* Georgi	0.85
Zingiberis rhizoma siccus	*Zingiber officinale*	0.03
Ginseng radix	*Panax ginseng C. A.* Meyer	0.45
Glycyrrhizae Radix	*Glycyrrhiza uralensis* Fischer	0.51
Jujubae fructus	*Zizyphus jujuba Mill*	0.34
Coptidis rhizoma	*Coptis chinensis* Franch	0.11
Zingiberis Rhizoma	*Zingiber officinale* Roscoe	0.28
Total		3.20

**Table 4:** BHSST used in this study. The analysis condition of 6-Gingerol, Glycyrrhizinic acid, Liquiritin, Isoliquiritigenin, Berberine, and Baicalin. FA: Formic Acid.

Time (min)	0.1% FA/Water (%)	0.1% FA/Acetonitrile (%)	Flow Rate (mL/min)
0	98	2	0.40
1.0	98	2	0.40
3.0	90	10	0.40
7.0	40	60	0.40
10.0	20	80	0.40
12.0	0	100	0.40
14.0	98	2	0.40
16.0	98	2	0.40

**Table 5:** The analysis condition of Ginsenoside Rg1.

Time (min)	Water (%)	Acetonitrile (%)	Flow Rate (mL/min)
0	85	15	0.40
1.0	85	15	0.40
14.0	70	30	0.40
15.0	68	32	0.40
16.0	60	40	0.40
17.0	45	55	0.40
19.0	45	55	0.40
21.0	10	90	0.40
22.0	10	90	0.40
23.0	85	15	0.40

**Table 6:** Contents of the seven marker compounds in the dried, powdered BHSST extract, measured by UPLC.

Compound (Marker)	Content in Extract (mg/g)	Calculated Marker Dose (mg/kg)
6-Gingerol	0.00106 ± 0.00020	0.00053 ± 0.00010
Glycyrrhizinic acid	0.01057 ± 0.00130	0.00529 ± 0.00065
Liquiritin	0.00353 ± 0.00015	0.00177 ± 0.00008
Isoliquiritigenin	0.00073 ± 0.00003	0.00037 ± 0.00002
Berberine	0.03300 ± 0.00075	0.01650 ± 0.00038
Baicalin	0.00111 ± 0.00027	0.00056 ± 0.00014
Ginsenoside Rb1	0.00153 ± 0.00013	0.00077 ± 0.00007

## Data Availability

The original data are available upon reasonable request to the corresponding author. The data are not publicly available due to privacy and ethical restrictions.

## Supplementary Materials

The following supporting information can be downloaded at: https://www.mdpi.com/article/10.3390/ph18081123/s1. Table S1: List of all active compound–target pairs and candidate target proteins for BHSST; Table S2: Additional molecular docking simulation results for active compounds of BHSST against TNF-α, PIK3CD, and PRKCD.
